# Cleavage of the extracellular domain of junctional adhesion molecule-A is associated with resistance to anti-HER2 therapies in breast cancer settings

**DOI:** 10.1186/s13058-018-1064-1

**Published:** 2018-11-20

**Authors:** Astrid O. Leech, Sri HariKrishna Vellanki, Emily J. Rutherford, Aoife Keogh, Hanne Jahns, Lance Hudson, Norma O’Donovan, Siham Sabri, Bassam Abdulkarim, Katherine M. Sheehan, Elaine W. Kay, Leonie S. Young, Arnold D. K. Hill, Yvonne E. Smith, Ann M. Hopkins

**Affiliations:** 1 Department of Surgery, Royal College of Surgeons in Ireland, RCSI Education and Research Centre, Beaumont Hospital, Dublin 9, Ireland; 2 Pathobiology Section, School of Veterinary Medicine, University College Dublin, Belfield, Dublin 4, Ireland; 3 National Institute for Cellular Biotechnology, Dublin City University, Collins Avenue, Dublin 9, Ireland; 4 Department of Pathology, McGill University, Faculty of Medicine, Department of Pathology, 1001 Decarie Blvd, Montreal, H4A 3J1 QC Canada; 5 Department of Oncology, McGill University, Faculty of Medicine, Department of Oncology, 1001 Decarie Blvd, Montreal, H4A 3J1 QC Canada; 6 Department of Pathology, Royal College of Surgeons in Ireland, RCSI Education and Research Centre, Beaumont Hospital, Dublin 9, Ireland

**Keywords:** JAM-A, Tight junction, HER2, Breast cancer, Drug resistance, HER2-targeted therapies, Trastuzumab, Herceptin, Lapatinib

## Abstract

**Background:**

Junctional adhesion molecule-A (JAM-A) is an adhesion molecule whose overexpression on breast tumor tissue has been associated with aggressive cancer phenotypes, including human epidermal growth factor receptor-2 (HER2)-positive disease. Since JAM-A has been described to regulate HER2 expression in breast cancer cells, we hypothesized that JAM-dependent stabilization of HER2 could participate in resistance to HER2-targeted therapies.

**Methods:**

Using breast cancer cell line models resistant to anti-HER2 drugs, we investigated JAM-A expression and the effect of JAM-A silencing on biochemical/functional parameters. We also tested whether altered JAM-A expression/processing underpinned differences between drug-sensitive and -resistant cells and acted as a biomarker of patients who developed resistance to HER2-targeted therapies.

**Results:**

Silencing JAM-A enhanced the anti-proliferative effects of anti-HER2 treatments in trastuzumab- and lapatinib-resistant breast cancer cells and further reduced HER2 protein expression and Akt phosphorylation in drug-treated cells. Increased epidermal growth factor receptor expression observed in drug-resistant models was normalized upon JAM-A silencing. JAM-A was highly expressed in all of a small cohort of HER2-positive patients whose disease recurred following anti-HER2 therapy. High JAM-A expression also correlated with metastatic disease at the time of diagnosis in another patient cohort resistant to trastuzumab therapy. Importantly, cleavage of JAM-A was increased in drug-resistant cell lines in conjunction with increased expression of ADAM-10 and -17 metalloproteases. Pharmacological inhibition or genetic silencing studies suggested a particular role for ADAM-10 in reducing JAM-A cleavage and partially re-sensitizing drug-resistant cells to the anti-proliferative effects of HER2-targeted drugs. Functionally, recombinant cleaved JAM-A enhanced breast cancer cell invasion *in vitro* and both invasion and proliferation in a semi-*in vivo* model. Finally, cleaved JAM-A was detectable in the serum of a small cohort of HER2-positive patients and correlated significantly with resistance to HER2-targeted therapy.

**Conclusions:**

Collectively, our data suggest a novel model whereby increased expression and cleavage of JAM-A drive tumorigenic behavior and act as a biomarker and potential therapeutic target for resistance to HER2-targeted therapies.

**Electronic supplementary material:**

The online version of this article (10.1186/s13058-018-1064-1) contains supplementary material, which is available to authorized users.

## Background

HER2 was originally described to be amplified in about 30% of human breast cancers [1]. Refinements in analytical techniques [2] now estimate the rate of HER2 protein overexpression to be 13–23% [3, 4]. Multiple complex pathways interact through the HER2 receptor, specifically in driving anti-apoptotic and pro-proliferative signaling through pathways including phosphoinositide-3-kinase (PI3K) and mitogen-activated protein kinase (MAPK) [5]. HER2-targeted therapies [6] have dramatically improved survival outcomes for HER2-positive patients, and new combinations continue to evolve in tandem with the development of new kinase inhibitors, monoclonal antibodies, and antibody-drug conjugates. However, although combined treatment has provided significant clinical benefit, many patients treated with dual anti-HER2 therapy experience either *de novo* or acquired drug resistance [7–12]. Therefore, elucidating novel mechanisms of resistance to anti-HER2 therapy is an important element in the search for new drug targets. This study presents evidence of a novel role for the adhesion protein JAM-A in resistance to anti-HER2 treatments.

JAM-A (also called the F11 receptor; gene name F11R) is a tight junction protein expressed principally by epithelial and endothelial cells but also by circulating leukocytes and platelets. It is composed of two extracellular immunoglobulin-like domains in its N-terminal tail, a single transmembrane region, and a short C-terminal cytosolic tail [13]. JAM-A has been shown to regulate several pro-tumorigenic processes, including proliferation, migration, invasion, and cell survival [14–17]. Although its role has been best studied in breast cancer [14, 16, 18, 19], evidence that JAM-A contributes to glioblastoma, nasopharyngeal, gastric, and lung cancer is also emerging (recently reviewed in [20]).

We previously reported a correlation between JAM-A and HER2 expression in aggressive breast tumors [18, 19] and showed that JAM-A regulates HER2 protein expression [18]. This led us to hypothesize that JAM-dependent stabilization of HER2 levels and signaling could identify a population of HER2-positive patients at risk of developing resistance to HER2-targeted therapies. Accordingly, we set out to investigate whether JAM-A overexpression correlated with resistance to HER2-targeted therapies in cell line models and patient tissues and to understand potential mechanisms of crosstalk that might drive drug resistance. Our findings present the first evidence that JAM-A overexpression and its cleavage by ADAM enzymes are associated with anti-HER2 drug resistance and with aggressive phenotypes in patients with HER2-positive breast cancer. Furthermore, we show for the first time that cleaved JAM-A may act as a novel ligand to drive cellular invasion events in breast cancer. This suggests future potential for co-targeting JAM-A and HER2 in the setting of HER2-positive patients resistant to HER2-targeted therapies.

## Methods

### Breast cancer cell culture and drug treatments

BT-474 trastuzumab-resistant (BT-474-Tr) cells, SK-BR-3 lapatinib-resistant (SK-BR-3-L) cells, and their corresponding drug-sensitive breast cancer cell lines (BT-474-Sens and SK-BR-3-Sens, respectively) were a kind gift from Alex Eustace (Royal College of Surgeons in Ireland) and Norma O’Donovan (Dublin City University) [21–23]. All cell lines were maintained in RPMI-1640 medium (Sigma-Aldrich, St. Louis, MO, USA) supplemented with 10% fetal bovine serum (FBS), 50 U/mL penicillin, and 50 μg/mL streptomycin. BT-474-Tr cells were cultured in 100 μg/mL trastuzumab (St. James’ Hospital Pharmacy) while SK-BR-3-L cells were cultured in 250 nM lapatinib (Sequoia Research Products, Pangbourne, UK). Drug resistance was verified via cellular viability assays (whereby 100 μg/mL trastuzumab reduced the viability of BT-474-Sens cells by 30% but was without effect on BT-474-Tr cell viability). Correspondingly, 250 nM lapatinib treatment reduced the viability of SK-BR-3-Sens cells by about 50% while that of SK-BR-3-L cells was reduced by less than 15% compared with vehicle-treated conditions (Additional file 1). All cell lines were maintained in a humidified incubator at 37 °C and 5% CO_2_ and were assessed for mycoplasma contamination every 3–4 months. DNA from all cell lines was sequenced yearly via short tandem repeat genotyping to confirm their identity.

### Transfections

Cells were transfected with 25 nM control small interfering RNA (siRNA) (siNEG, D-001210-01-05, Dharmacon, Lafayette, CO, USA), JAM-A siRNA (SASI_Hs01_00048785, Sigma-Aldrich), or siRNA to ADAM-10 ± siRNA to ADAM-17 (D-004503-01 and D-003453-01 respectively, Dharmacon). JAM-A silencing experiments were replicated by using an alternative siRNA specific for a different region of JAM-A mRNA (GGGGGUC GCAGGAAUCUGUU, Dharmacon; Additional file 2). Transfections were carried out using Lipofectamine 2000 (Invitrogen, Carlsbad, CA, USA) in accordance with the instructions of the manufacturer.

### Cell viability assays

Cellular viability was measured by 3-(4,5-dimethylthiazol-2-yl)-2,5-diphenyltetrazolium bromide (MTT) assays. Cells (1.5 × 10^3^) were plated in triplicate wells of 96-well plates and transfected the following day with 25 nM control siRNA or JAM-A siRNA. In select experiments, cells were treated with either vehicle control (dimethyl sulfoxide (DMSO), 0.3% vol/vol) or the ADAM inhibitor GI254023X (GI25; 12 μg/mL; Sigma-Aldrich SML0789); 24 h later, cells were treated with anti-HER2 therapy for 72 h (100 μg/mL trastuzumab or 250 nM lapatinib). Sterile MTT reagent (0.5 mg/mL) was added to the cells and incubated in the dark for 5 h at 37 °C. Following aspiration of media/MTT reagent, 200 μL DMSO was added for 5 min at 37 °C, whereupon absorbance was measured at 540 nm using a VICTOR™ X3 Multilabel Plate Reader (PerkinElmer, Waltham, MA, USA).

### Electrophoresis and Western blot analysis

Cells were washed with ice-cold phosphate-buffered saline (PBS) and scraped in lysis buffer (0.1 M KCl, 2.5 mM NaCl, 3.5 mM MgCl_2_, 10 mM HEPES pH 7.4, 1% Triton-X100, protease and phosphatase inhibitor cocktails; Sigma-Aldrich). Cells were lysed via trituration, extracts were centrifuged at 1500*g* for 5 min at 4 °C, and supernatants were stored at −20 °C. Protein content was quantified via bicinchoninic acid assay (BCA) (Thermo Fisher Scientific, Waltham, MA, USA), and 15 μg protein/lane was subjected to reducing SDS-PAGE, transferred to nitrocellulose membrane at 100 V for 75 min, and immunoblotted with the following antibodies: JAM-A (mouse, Becton Dickinson 612120; Becton Dickinson, Franklin Lakes, NJ, USA), HER2 (mouse, Becton Dickinson 610161), phosphorylated Akt (rabbit, Cell Signaling 9271; Cell Signaling Technology, Danvers, MA, USA), phosphorylated ERK (rabbit, Cell Signaling 4695), epidermal growth factor receptor (EGFR) (rabbit, Santa-Cruz sc-03; Santa-Cruz Biotechnology, Dallas, TX, USA), ADAM10 (rabbit, Cell Signaling 14194), ADAM17 (rabbit, Cell Signaling 3976), or β-actin (rabbit, Abcam ab8227; Abcam, Cambridge, UK). Secondary antibodies were all horseradish peroxidase–labeled and generated in goats. It is noteworthy that JAM-A, being an N-glycosylated protein, has different levels of glycosylation and therefore an expected level of variation in its appearance on Western blots. As a loading control for the cleaved JAM-A experiments, some electroblotted membranes were stained with 0.1% (wt/vol) Ponceau S in 5% acetic acid (Sigma-Aldrich) and then de-stained by washing in 1X Tris-buffered saline.

### RT-qPCR analysis

Cells were seeded at 5 × 10^4^ cells per well in 24-well plates for quantitative polymerase chain reaction (qPCR) analysis. At 72 h, RNA was harvested using Tri-Reagent (Sigma-Aldrich). Reverse transcription was carried out with 1 μg RNA using a QuantiTect Reverse Transcription Kit (205311, Qiagen, Hilden, Germany). The resulting cDNA was subjected to qPCR using the LightCycler 480 SYBR Green I Master (04707516001, Roche, Basel, Switzerland). Levels of JAM-A and EGFR mRNA were assessed in all cell lines using the following primers: JAM-A, forward: CTCTCAGTCCCCTCGCTGTA and reverse: AATGCCAGGGAGCACAACAG; EGFR, forward: TGCCATCCAAACTGCACCTA and reverse: TCTTAGGCCCATTCGTTGGAC, RPLP0, forward: GGCAGCATCTACAACCC TGA and reverse: AACATTGCGGACACCCTCC. Analysis was carried out using the double delta cycle threshold (∆∆Ct) method relative to RPLP0 as a reference housekeeping gene for standardization.

### Cleaved JAM-A (cJAM-A) detection

Cells were plated at 1.5 × 10^5^ cells per well in six-well plates; 48 h later, cells were washed in PBS and incubated for 72 h in serum-free media containing either vehicle control (DMSO, 0.3% vol/vol) or the ADAM inhibitor GI254023X (12 μg/mL). Media was centrifuged to remove dead cells and then concentrated using Amicon Ultra-2 Centrifugal Filter Spin Columns with a molecular weight cutoff of 3 kDa (UFC200324, Merck Millipore, Burlington, MA, USA). Protein concentration was quantified by BCA, and cJAM-A was detected by Western blot analysis. For the detection of cJAM-A in serum samples of patients with breast cancer, a JAM-A sandwich enzyme-linked immunosorbent assay (ELISA) was carried out in accordance with the instructions of the manufacturer (ELH-JAMA-1, Ray Biotech, Atlanta, GA, USA). In brief, duplicate serum samples (or recombinant human JAM-A standards) were incubated on microplate wells containing immobilized anti-human JAM-A antibody. After wash steps, biotinylated anti-human JAM-A was used as the detection antibody, followed by streptavidin-coupled horseradish peroxidase and a 3,3,5,5′-tetramethylbenzidine substrate solution. Optical densities of standards versus samples were measured at 450 nm using a VICTOR™ X3 Multilabel Plate Reader. Serum levels of cJAM-A were extrapolated from a standard curve generated using recombinant JAM-A. Serum samples were obtained at the time of routine follow-up appointments from 20 HER2-positive female breast cancer patients with invasive ductal carcinoma who had been treated with trastuzumab in Beaumont Hospital (Dublin, Ireland). Of the population, nine patients had not developed disease recurrence at the time their blood was sampled for cJAM-A analysis (and were considered therapy-responsive) whereas 11 patients had developed a disease recurrence and were deemed therapy-resistant. Patient demographics are summarized in Fig. 4B.

### Invasion assays

SK-BR-3-Sens cells (1 × 10^5^) were plated per well in six-well plates; 24 h later, cells were incubated for 72 h in serum-free media containing vehicle control (PBS) or specified concentrations of recombinant cleaved JAM-A (rcJAM-A; ab151859, Abcam). After trypsinization, 2 × 10^5^ cells from each condition were inserted into the top chamber of a Matrigel invasion assay in serum-free media containing either PBS or rcJAM-A; 5% FBS in the lower chamber was used as a chemoattractant; 16 h later, cells that had not invaded the Matrigel were scrubbed off the top surface of the membrane using Q-tips while invaded cells were fixed and stained with 0.5% crystal violet. An image was taken in each quadrant of the membrane at 20× magnification, and the number of invaded cells within each field was counted in a blinded fashion by an independent party.

### Chorioallantoic membrane assay

To examine potential pro-invasive/pro-tumorigenic effects of rcJAM-A treatment in a semi-*in vivo* system, the chicken embryo chorioallantoic membrane (CAM) model was used. A window was exposed in the shells of fertilized hen eggs, and on day 9 of embryonic development, 2 × 10^6^ SK-BR-3 breast cancer cells in 100 μL of 50% (vol/vol) Matrigel were seeded in the center of a sterile silicon ring introduced onto the CAM. The developing tumors were treated from days 10–13 (inclusive) with 40 ng rcJAM-A (equivalent of 2 mL 1 ng/mL treatment of 1 × 10^5^ cells). On day 14 of development, tumor xenografts and the surrounding CAM were excised, measured, and paraffin-embedded and sections were stained with hematoxylin/eosin, using immunohistochemical stains for pan-cytokeratin and Ki67 (performed using an auto-stainer in the Royal College of Surgeons in Ireland Pathology Department).

### Immunohistochemistry on material of patients with breast cancer

Immunohistochemistry (IHC) staining of JAM-A (using Abova mouse anti-JAM-A #H00050848-M01) was carried out in two separate cohorts of tissue samples of patients with breast cancer (in full accordance with ethical guidelines from each institution). The first cohort was an invasive breast cancer tissue microarray containing four cores each from 34 patients with HER2-positive breast cancer using samples from archival cases at Beaumont Hospital (Dublin, Ireland) from 2000 to 2008. The second cohort consisted of 77 full-face breast cancer tissue sections from patients with metastatic HER2-positive disease [24]. JAM-A staining was performed by the Royal College of Surgeons in Ireland Pathology Department using an automated IHC stainer. JAM-A expression in the breast tumor epithelium was principally membranous, but pools of cytoplasmic staining could also be observed. For the purposes of this study, we confined our analysis to membranous JAM-A expression intensity which was independently scored in a blinded fashion by one author (AOL) and a consultant breast histopathologist (KMS or EWK). Each core was assigned a value of 0, 1+, 2+, or 3+ (0 = no membrane staining; 1+ = incomplete membrane staining in more than 10% of the cells; 2+ = weak to moderate complete membrane staining in more than 10% of the cells; 3+ = strong and complete membrane staining in more than 10% of the cells).

### Statistical analysis

Averaged data from a minimum of *n* = 3 repeats of cell viability assays, Western blot experiments, reverse transcription–qPCR (RT-qPCR), and invasion assays were graphed along with standard error of the mean values. Two-tailed unpaired Student’s *t* tests were used to determine statistical significance between two treatment conditions. These were analyzed as either equal variance or unequal variance on the basis of results from F-test analyses. One-way analysis of variance with Tukey’s multiple comparison post test was used to determine statistical significance between more than two treatment conditions. STATA software was used to carry out chi-squared tests and Fisher’s two-tailed exact tests for correlation of JAM-A expression with clinicopathological factors in patient tissues. In all cases, *, ** and *** denote *P* <0.05, *P* <0.01 and *P* <0.001, respectively.

## Results

### Silencing JAM-A re-sensitizes BT-474-Tr and SK-BR-3-L cells to anti-HER2 therapy

**Fig. 1:**
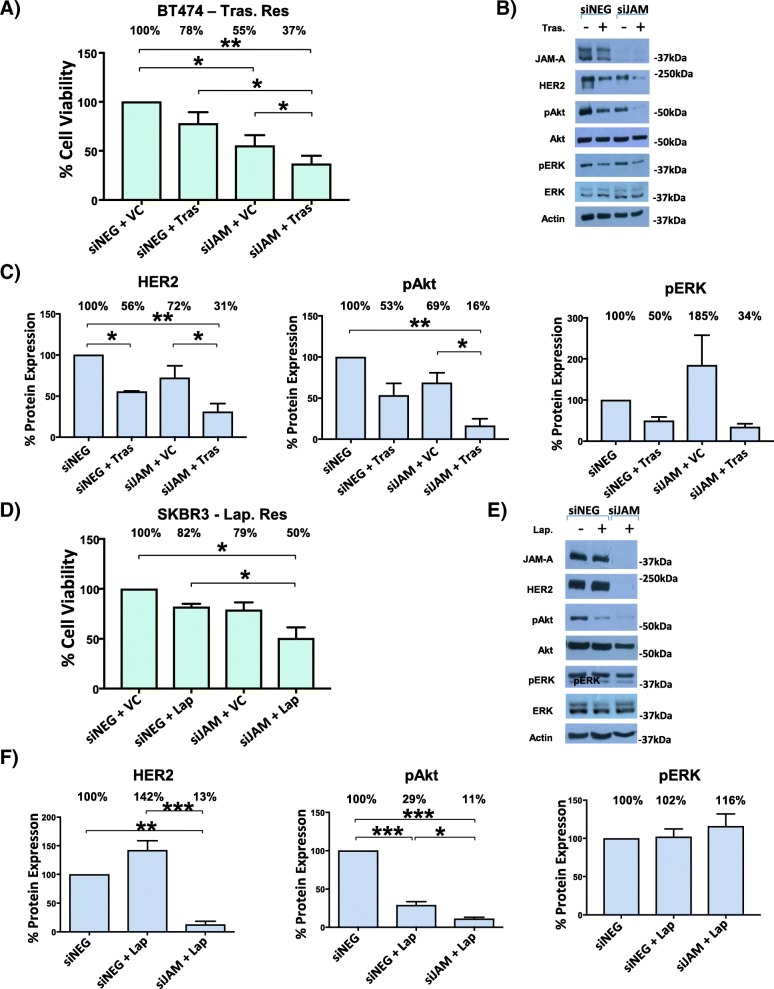
Silencing JAM-A re-sensitizes BT474 trastuzumab-resistant and SKBR3 lapatinib-resistant cells to anti-HER2 therapy. BT-474-trastuzumab-resistant cells (BT-474-Tr; A–C) or SK-BR-3-lapatinib-resistant cells (SK-BR-3-L; D–F) were plated in triplicate wells of 96-well plates (1500 cells per well) or in six-well plates (150,000 cells per well) and transfected the following day with 25 nM of control small interfering RNA (siRNA) (siNEG) or JAM-A siRNA; 24 h later, cells were treated with vehicle control (sterile nuclease-free water, 0.005% vol/vol or dimethyl sulfoxide, 0.0001% vol/vol), trastuzumab (100 μg/mL for BT-474-Tr cells), or lapatinib (500 nM for SK-BR-3-L cells); 72 h later, cell viability was measured via MTT (3-(4,5-dimethylthiazol-2-yl)-2,5-diphenyltetrazolium bromide) assay or cells were harvested for Western blot analysis. **(a)** Cell viability response of BT-474-Tr cells to trastuzumab treatment alone and combined with JAM-A knockdown. **(b)** Representative Western blot images for expression of HER2, pAkt, and pERK following specified treatments in BT-474-Tr cells. **(c)** Densitometry analysis of HER2, pAKT, and pERK expression in BT-474-Tr cells following specified treatments. Silencing JAM-A in addition to trastuzumab treatment reduced HER2 expression and Akt phosphorylation more than trastuzumab treatment alone in BT474-Tr cells. **(d)** Cell viability response of SK-BR-3-L cells to lapatinib treatment alone and combined with JAM-A knockdown. Silencing JAM-A expression in addition to anti-HER2 treatment was more effective at reducing resistant cell viability than anti-HER2 treatment alone. **(e)** Representative Western blot images for expression of HER2, pAkt, and pERK following specified treatments in SK-BR-3-L cells. **(f)** Densitometry analysis of HER2, pAKT, and pERK expression in SK-BR-3-L cells following specified treatments. Silencing JAM-A in addition to lapatinib treatment reduced Akt phosphorylation more than lapatinib treatment alone in SK-BR-3-L cells (*P <0.05, **P <0.01, ***P <0.001 by one-way analysis of variance with Tukey’s multiple comparison test, *n* = 3 independent experiments).

This work was founded upon observations that JAM-A overexpression correlates with aggressive disease phenotypes in patients with breast cancer and that JAM-A levels can regulate those of HER2 in breast cancer cells [18, 19]. As shown in Fig. 1, silencing JAM-A in combination with trastuzumab or lapatinib treatment enhanced the anti-viability properties of HER2-targeting drugs in both BT-474-Tr cells and SK-BR-3-L cells. Specifically, viability of BT-474-Tr cells was reduced to 78% ± 11% of control levels in response to trastuzumab treatment alone (*P* <0.01), whereas silencing JAM-A in addition to trastuzumab treatment further reduced viability to 37% ± 6% of control levels (Fig. 1A; *P* <0.001). Similarly, lapatinib treatment alone reduced the viability of lapatinib-resistant cells to 82% ± 3% of control levels whereas silencing JAM-A in addition to lapatinib treatment reduced cell viability to 50% ± 11% of control levels (Fig. 1D; *P* <0.05).

We then aimed to assess the effect of coincident JAM-A silencing/trastuzumab treatment on HER2 expression and its downstream signaling effectors in the PI3K and MAPK pathways. As shown in Fig. 1B–C, combined silencing of JAM-A and anti-HER2 treatment was more effective at reducing HER2 protein expression and Akt phosphorylation, but not ERK phosphorylation, than anti-HER2 treatment alone in BT-474-Tr cells. HER2 expression increased in SK-BR-3-L cells upon lapatinib treatment, but the combination of JAM-A silencing and lapatinib treatment significantly decreased HER2 expression relative to either control condition tested (Fig. 1E–F). Similar to the observed effects in BT-474-Tr cells, pAkt but not pERK levels were reduced upon drug treatment in SK-BR-3-L cells while concomitant JAM-A silencing further enhanced pAkt reductions. In both cell lines, total ERK2 (lower band) was expressed at higher levels than total ERK1 (upper band), but phosphorylation on ERK1 was dominant.

### Silencing JAM-A in trastuzumab- and lapatinib-resistant cells downregulates resistance-associated EGFR protein expression

**Fig. 2:**
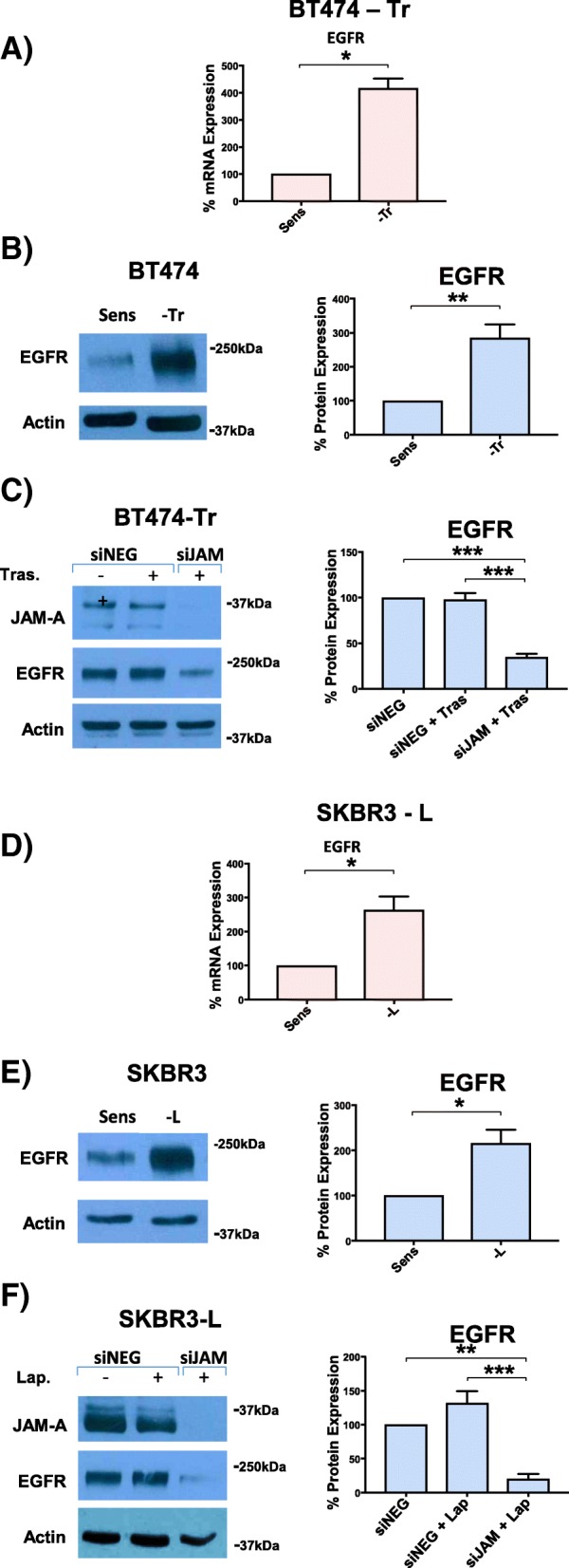
BT-474 trastuzumab-resistant cells and SK-BR-3 lapatinib-resistant cells express increased levels of epidermal growth factor receptor (EGFR) at both mRNA and protein level compared with their corresponding sensitive cells and JAM-A knockdown reduces EGFR expression. BT-474 trastuzumab-sensitive (BT-474-Sens), -resistant (BT-474-Tr), SK-BR-3 lapatinib-sensitive (SK-BR-3-Sens), and -resistant (SK-BR-3-L) cells were plated at 150,000 cells per well in six-well plates for Western blot analysis and at 50,000 cells per well in 24-well plates for quantitative polymerase chain reaction (qPCR) analysis. At 72 h, cells were harvested for Western blot and qPCR analysis. **(a)** qPCR analysis of EGFR mRNA expression in BT-474-Sens and BT-474-Tr cells. **(b)** Representative Western blot images and densitometric analysis for basal protein expression of EGFR in BT-474-Sens and BT-474-Tr cells (relative to actin). **(c)** Representative Western blot analysis and densitometric analysis for the effect of JAM-A knockdown and anti-HER2 treatment on EGFR protein expression in BT-474-Tr cells (relative to actin). **(d)** qPCR analysis of EGFR mRNA expression in SK-BR-3-Sens and SK-BR-3-L cells. **(e)** Representative Western blot images and densitometric analysis for basal protein expression of EGFR in SK-BR-3-lapatinib-sensitive and -resistant cells (relative to actin). **(f)** Representative Western blot analysis and densitometric analysis for the effect of JAM-A knockdown and anti-HER2 treatment on EGFR protein expression in SK-BR-3-L cells (relative to actin). BT-474-Tr and SK-BR-3-L cells expressed increased levels of EGFR at both mRNA and protein level compared with their corresponding sensitive cells, and JAM-A knockdown reduced this augmented EGFR expression. (*P <0.05 by equal variance unpaired *t* test, n = 3 independent experiments)

We next tested whether silencing JAM-A in trastuzumab- and lapatinib-resistant cells affected the expression of EGFR, another member of the HER family whose expressional upregulation has been frequently associated with drug resistance [25–27]. Accordingly, basal EGFR protein and mRNA expression were compared between drug-sensitive and -resistant cells. As illustrated in Fig. 2A–B, trastuzumab-resistant BT-474 cells expressed significantly higher levels of EGFR mRNA and protein than their trastuzumab-sensitive counterparts (*P* <0.05 and *P* <0.01, respectively). Interestingly, direct silencing of JAM-A was sufficient to significantly reduce EGFR protein expression in BT-474-Tr cells (Fig. 2C). These results were mirrored in SK-BR-3-L cells (Fig. 2D–F).

### JAM-A cleavage is augmented in cells with resistance to trastuzumab and lapatinib therapy

**Fig. 3:**
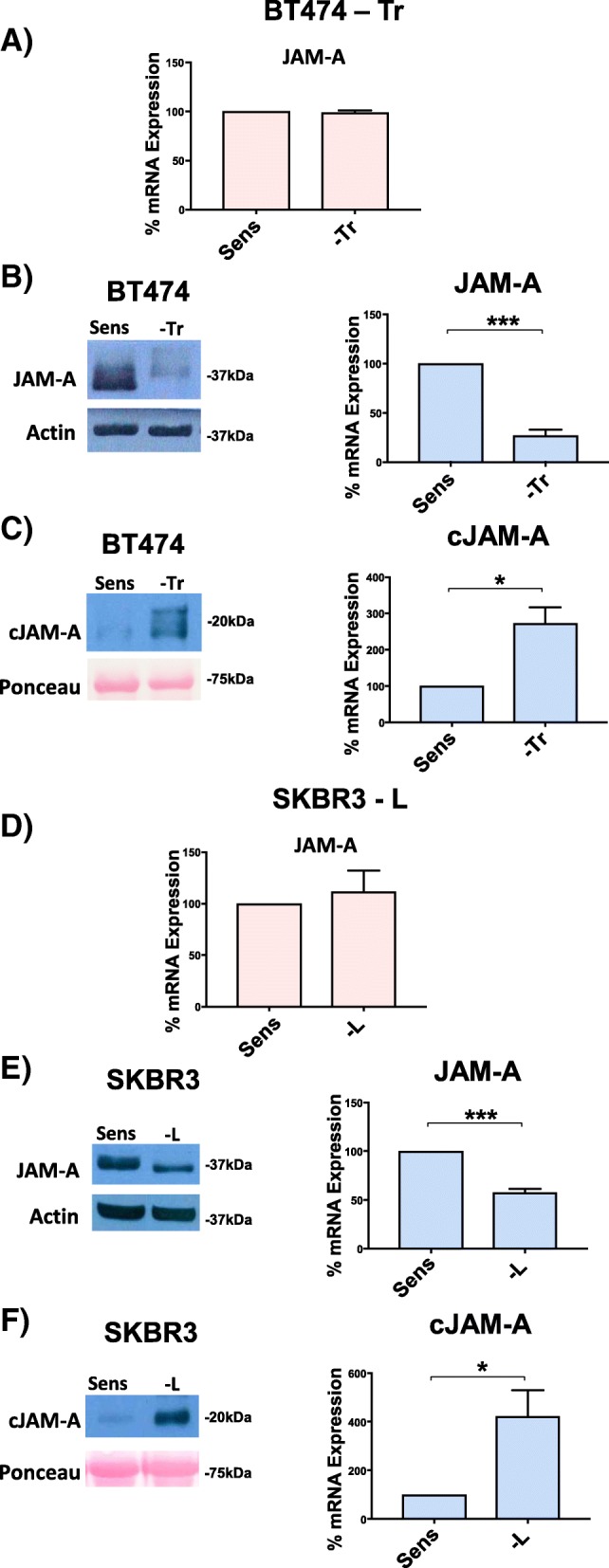
JAM-A cleavage is augmented in BT-474 trastuzumab-resistant and SK-BR-3 lapatinib-resistant cells compared with their corresponding drug-sensitive cell lines. BT-474 trastuzumab-sensitive (BT-474-Sens), -resistant (BT-474-Tr), SK-BR-3 lapatinib-sensitive (SK-BR-3-Sens), and -resistant (SK-BR-3-L) cells were plated at 150,000 cells per well in six-well plates for Western blot analysis and at 50,000 cells per well in 24-well plates for quantitative polymerase chain reaction (qPCR) analysis; 48 h later, cells designated for cleaved JAM-A (cJAM-A) detection were washed and serum-free media was added to the wells. At 72 h, conditioned media was centrifuged to exclude any floating cells and concentrated using Amicon Ultra-2 Centrifugal Filter Spin Columns, whereupon cells were harvested for Western blot and qPCR analysis. **(a)** qPCR analysis of JAM-A mRNA expression in BT-474-Sens and BT-474-Tr cells. **(b)** Representative Western blot images and densitometric analysis of basal JAM-A protein expression in BT-474-Sens and BT-474-Tr cells (relative to actin). **(c)** Representative Western blot images and densitometric analysis of basal cleaved JAM-A released from BT-474-Sens and BT-474-Tr cells (relative to Ponceau S). **(d)** qPCR analysis of JAM-A mRNA expression in SK-BR-3-Sens and SK-BR-3-L cells. **(e)** Representative Western blot images and densitometric analysis of basal JAM-A protein expression in SK-BR-3-Sens and SK-BR-3-L cells (relative to actin). **(f)** Representative Western blot images and densitometric analysis of basal cleaved JAM-A released from B SK-BR-3-Sens and SK-BR-3-L cells (relative to Ponceau S). JAM-A cleavage and release of cleaved JAM-A was significantly augmented in BT-474-Tr and SK-BR-3-L cells compared with their corresponding drug-sensitive cell lines. *P <0.05 by equal variance unpaired *t* test, *n* = 3 independent experiments

Having documented that JAM-A knockdown reduced the levels of key signaling molecules associated with driving tumorigenesis and drug resistance, we searched for a direct correlation between JAM-A expression levels and the acquisition of drug resistance. Contrary to expectations, RT-qPCR analysis showed no difference in JAM-A mRNA expression between cells resistant to anti-HER2 drugs and their corresponding drug-sensitive cell lines (Fig. 3A, D). More surprising yet, Western blot analysis revealed that resistance to anti-HER2 therapy in the same cell lines was associated with *loss* rather than gain of JAM-A protein expression in whole cell extracts (Fig. 3B, E). This was at odds with our earlier results demonstrating that silencing JAM-A re-sensitized trastuzumab- and lapatinib-resistant cells to anti-HER2 therapy. We therefore questioned whether resistance-associated reductions in JAM-A protein expression reflected loss of a specific pool of the protein from those cells. Because the antibody used for Western blot analysis targeted the extracellular domain of JAM-A, we questioned whether this domain was being shed from the drug-resistant cells and washed away prior to harvesting cells for electrophoresis. Analysis of the culture medium bathing both drug-resistant and drug-sensitive cells accordingly revealed significantly higher levels of JAM-A in the extracellular milieu of BT-474-Tr and SK-BR-3-L cells compared with their corresponding drug-sensitive cell lines (Fig. 3C, F). This suggested that JAM-A protein was being shed from the cells. We have termed this extracellularly-released JAM-A product “cleaved JAM-A” (cJAM-A).

### Increased levels of cleaved JAM-A in serum of patients with HER2-positive breast cancer may correlate with development of resistance to anti-HER2 therapy

We then questioned whether cJAM-A could be detected in the serum of patients with HER2-positive breast cancer and, if so, whether its presence correlated with drug resistance.

**Fig. 4:**
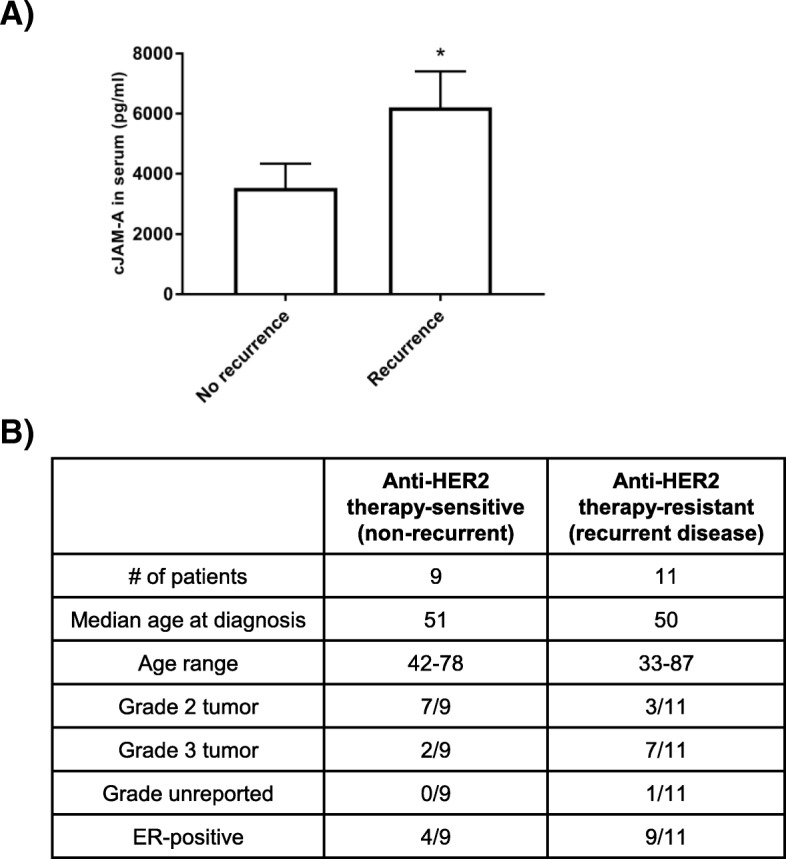
Cleaved JAM-A levels are increased in the serum of patients with HER2-positive breast cancer resistant to HER2-targeted therapy. **(a)** Enzyme-linked immunosorbent assays were used to measure levels of cleaved soluble JAM-A (cJAM-A) in the serum of HER2-positive invasive ductal breast cancer patients who responded to trastuzumab therapy (*n* = 9) and those who developed a disease recurrence while receiving trastuzumab (*n* = 11). *P <0.05 by unequal variance unpaired one-tailed *t* test. **(b)** Patient characteristics

To this end, serum samples were obtained from a pilot population of patients with HER2-positive breast cancer treated with anti-HER2 therapy (*n* = 20, of whom 11 had developed a disease recurrence by the time of sampling). Those patients were deemed resistant to HER2-targeted therapy, whereas the 9 out of 20 who had not developed a recurrence were deemed treatment-sensitive. cJAM-A levels detected by ELISA in the serum ranged from about 350 to 16,000 pg/mL, and (as shown in Fig. 4A) levels in therapy-resistant patients were significantly higher.

### ADAM10 and ADAM17 protein expression is augmented in BT-474-Tr and SK-BR-3-L cells and mediates JAM-A cleavage

**Fig. 5:**
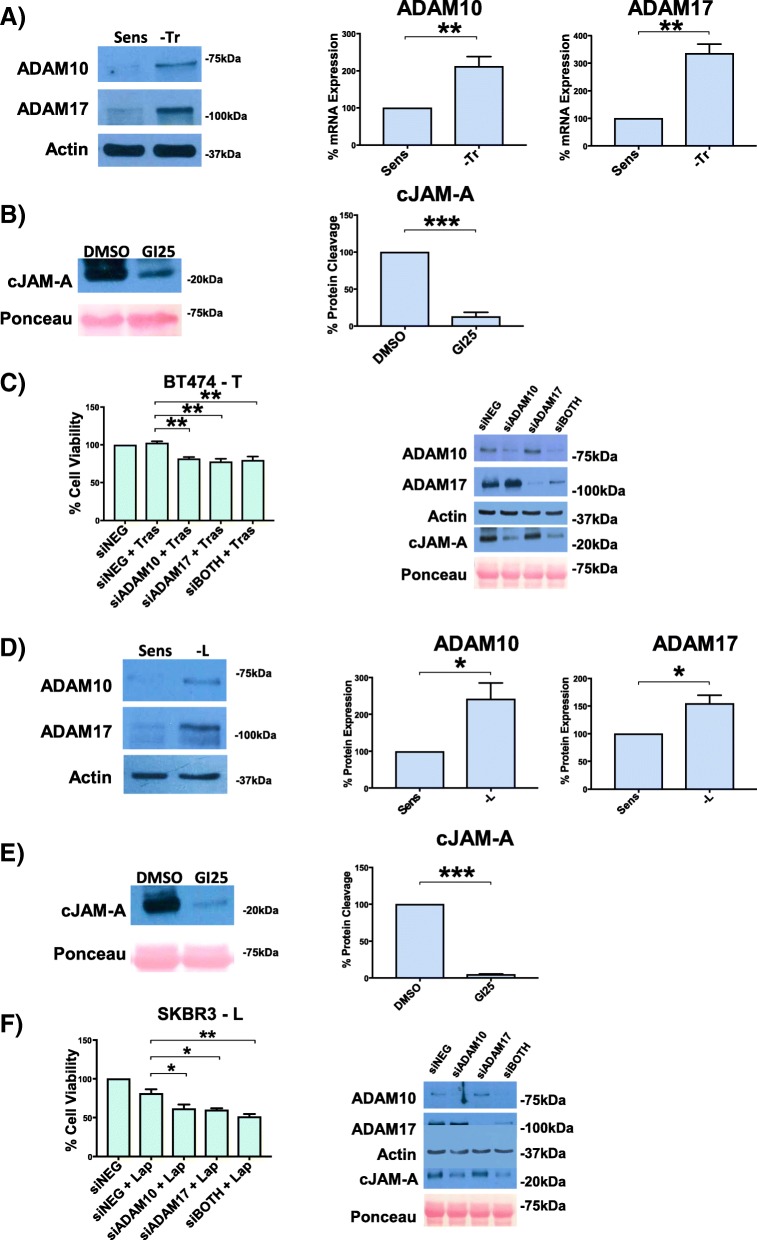
Enhanced protein expression of ADAM10 and ADAM17 and accompanying cleavage of JAM-A are associated with acquired resistance to trastuzumab and lapatinib. BT-474 trastuzumab-sensitive (BT-474-Sens), -resistant (BT-474-Tr), SK-BR-3 lapatinib-sensitive (SK-BR-3-Sens), and -resistant (SK-BR-3-L) cells were plated at 150,000 cells per well in six-well plates for Western blot analysis and at 1500 cells per well in 96-well plates for MTT (3-(4,5-dimethylthiazol-2-yl)-2,5-diphenyltetrazolium bromide) analysis; 24 h later, cells were transfected with 25 nM control small interfering RNA (siRNA), ADAM10 siRNA, ADAM17 siRNA, or a combination. The following day, BT-474-Tr cells were treated with vehicle control (sterile nuclease-free water, 0.5% vol/vol) or 100 μg/mL trastuzumab while SK-BR-3-L cells were treated with vehicle control (dimethyl sulfoxide, 0.002% vol/vol) or 250 nM lapatinib; 72 h later, cell viability was measured via MTT assay and cells plated for Western blot analysis were harvested. **(a)** Representative Western blot images and densitometric analysis of basal protein expression of ADAM10 and ADAM17 in BT-474-Tr cells (relative to actin). **(b)** Representative Western blot images and densitometric analysis of cleaved JAM-A (cJAM-A) release following ADAM10 and ADAM17 inhibition using GI254023X (GI25) in BT-474-Tr cells (relative to Ponceau S). **(c)** Cell viability of BT-474-Tr cells following knockdown of ADAM10 or ADAM17 (or both) and subsequent anti-HER2 treatment. **(d)** Representative Western blot images and densitometric analysis of basal protein expression of ADAM10 and ADAM17 in SK-BR-3-L cells (relative to actin). **(e)** Representative Western blot images and densitometric analysis of cleaved JAM-A (cJAM-A) release following ADAM10 and ADAM17 inhibition using GI254023X (GI25) in SK-BR-3-L cells (relative to Ponceau S). **(f)** Cell viability of SK-BR-3-L cells following knockdown of ADAM10 or ADAM17 (or both) and subsequent anti-HER2 treatment. ADAM10 and ADAM17 protein expression was upregulated in cells with acquired resistance to trastuzumab and lapatinib, causing increased JAM-A cleavage. Silencing ADAM10 or ADAM17 (or both) reduced JAM-A cleavage and partially re-sensitized BT-474-Tr and SK-BR-3-L cells to anti-HER2 therapy. *P <0.05, **P <0.01 by equal variance unpaired *t* test, n = 3 independent experiments

To elucidate potential reasons for increased JAM-A cleavage in trastuzumab- and lapatinib-resistant cellular models, we tested whether this reflected elevated expression of two metalloproteases previously shown to cleave JAM-A (ADAM10 and ADAM17 [28]). As shown in Fig. 5A,D, BT-474-Tr and SK-BR-3-L cells (respectively) expressed significantly higher levels of both ADAM10 and ADAM17 compared with their corresponding drug-sensitive cell lines. Furthermore, pharmacological inhibition of these proteases using GI254023X (GI25) dramatically reduced the release of cJAM-A from BT-474-Tr (Fig. 5B) and SK-BR-3-L (Fig. 5E) cells. Also, successful silencing of ADAM10, which dramatically reduced JAM-A cleavage, was associated with reductions in the viability of anti-HER2 drug-treated BT-474-Tr (Fig. 5C) and SK-BR-3-L (Fig. 5F) cells. Successful silencing of ADAM17 had much less effect on cJAM-A levels but still significantly reduced cell viability in the presence of either anti-HER2 drug, implying that ADAM17 is less important than ADAM10 in generating cJAM-A and that there are likely to be other ADAM targets of importance besides JAM-A in regulating cell viability. There was no additive reduction in cell viability when both ADAMs were simultaneously silenced in the presence of either anti-HER2 drug. Since the concentrations of each individual siRNA were halved for use in combination experiments and the capacity of siADAM10 to reduce cJAM-A expression would be diluted by the weaker one of siADAM17, it is possible that the lack of an additive reduction on cell viability reflects incomplete inhibition of JAM-A cleavage. However, ADAM inhibition with GI25 treatment only reduced cellular viability to a small (yet statistically significant) degree independently of treatment status with anti-HER2 drugs (Additional file 3) despite substantial inhibition of JAM-A cleavage under similar conditions (Fig. 5B, F). As JAM-A silencing exerted greater reductions in cell viability (Fig. 1A, D) than inhibition of JAM-A cleavage, this may suggest that the membrane-tethered tail of JAM-A remaining after JAM-A cleavage is a more important regulator of cell viability than cJAM-A itself.

### Cleaved JAM-A promotes an invasive phenotype *in vitro* and in a semi-*in vivo* model

**Fig. 6:**
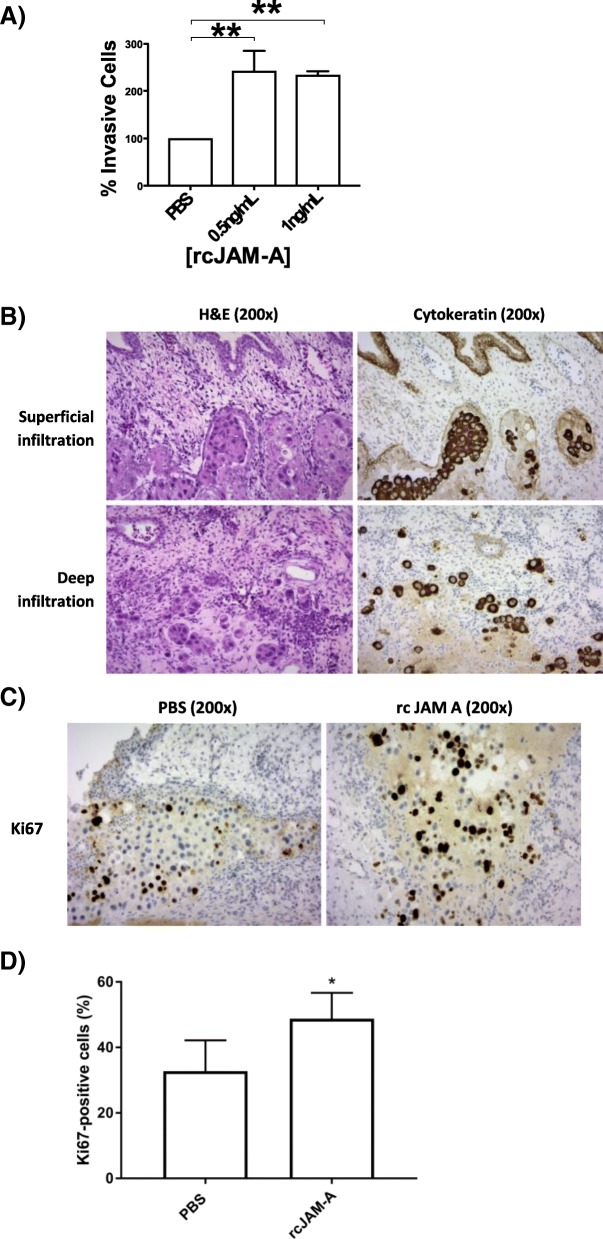
Recombinant cleaved JAM-A treatment promotes invasion of drug-sensitive SK-BR-3 cells *in vitro* and a semi-*in vivo* model. SK-BR-3–sensitive (SK-BR-3-Sens) cells (100,000) were plated per well in six-well plates for invasion assays; 24 h later, cells were treated in serum-free media with vehicle control (phosphate-buffered saline, or PBS) or specified concentrations of recombinant cleaved JAM-A (rcJAM-A; ab151859, Abcam); 72 h later, 200,000 cells from each condition were inserted into the top chamber of a Matrigel invasion assay in serum-free media with PBS or rcJAM-A treatment; 5% fetal bovine serum in the lower chamber was used as a chemoattractant; 16 h later, non-invaded cells were scrubbed off, and invaded cells were fixed and stained with 0.5% crystal violet. An image was taken in each quadrant of the membrane at 20× magnification, and invaded cells were counted by an independent party. **(a)** Quantification of drug-sensitive SK-BR-3 cellular invasion in response to recombinant cleaved JAM-A treatment. *P <0.05 by one-way analysis of variance with Tukey’s multiple comparison test, *n* = 4 independent experiments. **(b–d)** SK-BR-3-Sens cells (2 × 10^6^) were seeded on the chorioallantoic membrane (CAM) of chick embryos in a silica ring. The following day, tumor xenografts were treated with 40 ng rcJAM-A or PBS daily for 4 days. The tumor and surrounding CAM were then excised, fixed in 4% paraformaldehyde overnight, and paraffin-embedded. (B) In the majority (71%) of rcJAM-A treated xenografts, invasion of neoplastic cells deep into the intermediate mesodermal layer of the CAM was observed, whereas this pattern was seen in only 43% of PBS-treated tumors. Photomicrographs show representative examples of superficial (neoplastic cells only infiltrating the chorionic epithelium) and deep (neoplastic cells present within the intermediate mesodermal layer) tumor invasion. Hemotoxylin and eosin (H&E) and pan-cytokeratin, 200× magnification. (C, D) Tumor xenografts were stained for the proliferation marker Ki67. The proliferation index (percentage Ki67-positive cells per 500 tumor cells) was significantly higher in rcJAM-A–treated tumor xenografts than in controls. Representative microscopic images are shown of low and high Ki67 expresssion in controls and treated tumors. Ki67 (Mib 1) 200× magnification. *P <0.05 by two-tailed equal variance unpaired *t* test

To dissect the potential pro-tumorigenic functions of cJAM-A in more detail, a recombinant fragment of the JAM-A extracellular domain was used to mimic cJAM-A (rcJAM-A). Specifically, the aim was to examine whether rcJAM-A application to drug-sensitive (not drug-resistant) cells would be sufficient to induce functional behaviors associated with increased tumorigenicity. Because results to date had been similar in BT-474-Sens and SK-BR-3-Sens cells, SK-BR-3-Sens cells were selected as the representative model. Treatment concentrations of rcJAM-A for SK-BR-3-sens cells (0.5–1 ng/mL) were chosen on the basis of basal cJAM-A levels released by SK-BR-3-L cells (data not shown). Short-term rcJAM-A treatment had no effect on the viability of drug-sensitive cell lines in response to anti-HER2 drugs (Additional file 4), which was unsurprising given the earlier point that inhibition of JAM-A cleavage had less effect on viability than loss of *total* JAM-A. Similarly, rcJAM-A did not promote the colony-forming abilities of drug-sensitive cells (Additional file 4). However, as illustrated in Fig. 6a, rcJAM-A treatment (0.5 ng/mL) dramatically increased the invasive capacity of SK-BR-3–sensitive cells through Matrigel in modified Boyden chamber assays (by 143% ± 43%) compared with vehicle control-treated cells (*P* <0.01). A significant elevation of invasive capacity (by 134% ± 8%) was also observed upon treatment with 1 ng/mL rcJAM-A relative to control conditions (*P* <0.05).

To determine whether rcJAM-A treatment had pro-invasive or pro-tumorigenic effects in a higher-order experimental model, a semi-*in vivo* approach using chick embryo CAM assays was adopted. The CAM is a vascular membrane found in chick eggs which helps the embryo exchange gases and handles liquid waste, thus serving the functions of a placenta for the growing chick embryo. Owing to the presence of blood vessels to facilitate gas exchange and extracellular matrix proteins, the CAM model mimics the tumor micro-environment [29] and has become an established semi-*in vivo* xenograft system to assess tumor progression [29–32]. Implanted xenograft tumors that developed following treatment with rcJAM-A were grossly larger in size compared with those treated with vehicle control (data not shown). Tumor invasion of the CAM was assessed as either superficial (chorionic epithelium not penetrated) or deep (infiltration of cells into the intermediate mesodermal layer) on hemotoxylin and eosin (H&E)– and pan-cytokeratin–stained sections. The majority of rcJAM-A–treated tumors (71%) showed deep tumor invasion, whereas only 43% of the controls showed this pattern (Fig. 6B). In order to further assess the pro-tumorigenic effects of rcJAM-A, sections were stained with the proliferation marker Ki-67 (Mib1; Fig. 6C). The proliferation index was also calculated for rcJAM-A–treated and control tumors on one representative cross-section each by quantifying Ki-67 expression in 500 tumor cells infiltrating the CAM interface (epithelium and intermediate mesodermal layer). A small but significant increase in the proliferation index was observed in rcJAM-A–treated tumors compared with controls (Fig. 6D).

**Fig. 7:**
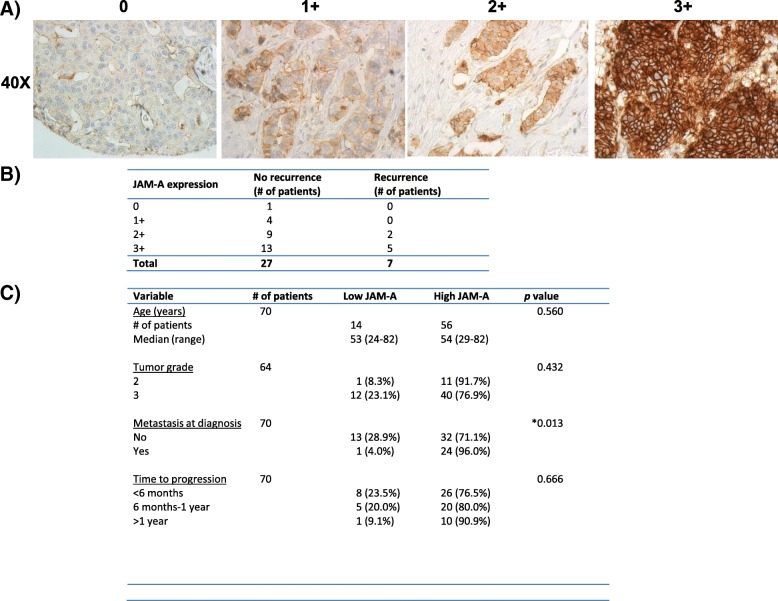
JAM-A overexpression correlates with disease recurrence or metastasis in two cohorts of patients with HER2-positive breast cancer. A tissue microarray composed of breast tissue cores from 34 HER2-positive carcinomas and full-face sections from 70 HER2-positive breast cancer patients with metastatic disease was analyzed for JAM-A expression. Tissue was scored 0, 1+, 2+, or 3+ on the basis of intensity of membranous JAM-A staining **(a)**. JAM-A was overexpressed in 7 out of 7 tumors of the 34 HER2-positive patients who developed disease recurrence **(b)**. JAM-A overexpression also significantly correlated with the presence of metastatic disease at diagnosis in 70 HER2-positive breast carcinoma full-face sections **(c)**. *P <0.05 by two-tailed Fisher’s exact test

In summary, our findings have indicated that targeting JAM-A in addition to HER2 betters anti-HER2 drugs alone in reducing cell viability and tumorigenic signaling and invasion and that cJAM-A may act as a biomarker of drug resistance in patients. Because local invasion is an early pre-requisite for metastasis to occur, we sought to directly test a link between JAM-A overexpression, metastasis, and resistance to anti-HER2 therapy. Accordingly, JAM-A expression was first immunohistochemically analyzed in a small tissue microarray from 34 HER2-positive breast carcinomas (for representative images of the semi-quantitative scoring system used, see Fig. 7A). As shown in Fig. 7B, JAM-A was overexpressed (2+ or 3+ staining intensity) in the breast tumors of 7 out of 7 patients who developed disease recurrence following HER2-targeted therapy (in comparison with 22 out of 27 patients whose disease had not recurred up to the time point of analysis). A strong correlation between JAM-A expression and the presence of metastasis in HER2-positive patients was also noted in a separate cohort of patients (*n* = 70). As shown in Fig. 7C, JAM-A was overexpressed (2+ or 3+ staining intensity) in 80% (56 out of 70) of tumors from patients with metastatic HER2-positive breast carcinoma. In addition, there was a significant association between JAM-A overexpression and metastasis, as 96% of patients who had metastatic disease at diagnosis overexpressed JAM-A in their primary tumors (*P* <0.05). There were no significant correlations between JAM-A expression and time to progression, which is perhaps unusual given that the patients who progressed in any of the listed time frames were more likely to have high JAM-A expression rather than low JAM-A expression. The same was the case for higher tumor grade. The lack of statistical significance in both of these points may reflect patient heterogeneity within a relatively small cohort, illustrating the value of larger-scale studies preferably conducted at multiple sites.

## Discussion

Breast cancer is the most common cancer in women worldwide, accounting for about 20% of all cancers in women (http://www.wcrf.org/int/cancer-facts-figures/worldwide-data). The strong invasive potential of breast tumors poses a major clinical problem, as metastasis of the primary tumor to the liver, lung, and brain still carries a high risk of death [33, 34]. HER2-positive breast cancer is an aggressive form of the disease, accounting for about 20% of all breast cancers. Although there are several HER2-targeted therapies, therapeutic efficacy is often limited by *de novo* and acquired resistance [7–9]. While several factors have been proposed to predict clinical resistance to anti-HER2 therapies (reviewed by [35]), there is a need for continual research into new prognostic and predictive markers of resistance. Previous work in our lab has shown that the tight junction protein JAM-A is significantly associated with HER2 expression and poor clinical outcome in patients with breast cancer [18, 19]. In addition, insights that JAM-A positively regulates HER2 expression [18] led us to hypothesize that JAM-A expression could stabilize HER2-dependent signaling and play a role in resistance to anti-HER2 therapies.

We found that silencing JAM-A partially re-sensitized BT-474-Tr and SK-BR-3-L cells to anti-HER2 treatment. In addition, JAM-A knockdown combined with anti-HER2 treatment was more effective at downregulating HER2 expression and Akt phosphorylation than anti-HER2 treatment alone. Increased stability of HER2 expression has been shown to confer resistance to anti-HER2 therapies [36–38]. In addition, increased activation of the PI3K-Akt pathway through loss of *PTEN* or via *PIK3CA* mutations is frequently reported in conjunction with resistance [8, 39–41]. Our results have shown for the first time that JAM-A stabilizes EGFR expression in two drug-resistant cell line models, the upregulation of which is a characteristic of acquired resistance to anti-HER2 drugs [27, 42, 43]. As HER2 and EGFR can activate Akt (although HER2 would be its dominant activator in breast cancer) and Akt in turn can promote cell survival, the observed inhibition of cell growth following JAM-A knockdown in drug-resistant models may reflect downregulation of HER2, EGFR, and pAkt. It is interesting to note that, in our study, pAkt levels went down in lapatinib-treated drug-resistant cells in the absence of reductions in either EGFR or HER2. However, because lapatinib treatment caused some JAM-A downregulation and JAM-A levels have been shown to positively correlate with Akt phosphorylation [44], it is intriguing to speculate that JAM-A levels might independently regulate Akt phosphorylation in certain cancer settings. In fact, *in vivo* murine studies have confirmed an involvement for JAM-A in breast tumor proliferation and shown that an anti-JAM-A monoclonal antibody decreases growth of murine breast tumor xenografts [17]. JAM-A has also been shown to promote proliferation in gastric cancer [45]. In addition, other *in vivo* studies have highlighted a role for JAM-A expression in opposing apoptosis, another key function of pAkt. Specifically, JAM-A null mice were shown to have increased apoptosis and therefore smaller tumors than JAM-A–positive mice [16]. Taken together, our findings suggest that JAM-A expression promotes HER2 and EGFR expression as well as downstream Akt activation and survival of cells with acquired resistance to anti-HER2 therapy.

Subsequent experimentation revealed a novel association between ADAM-mediated JAM-A cleavage and resistance to anti-HER2 therapies. Interestingly, recent studies have reported an association between upregulation of ADAM10 and ADAM17 and acquired resistance to trastuzumab and lapatinib in patients with HER2-positive breast cancer [46–49, 53]. However, despite being a target of these proteases (in our study, principally ADAM10), the role of JAM-A has never been considered in that setting. It is intriguing to speculate that JAM-A extracellular cleavage enhances tumorigenesis by alleviating the intercellular adhesive functions of JAM-A (which are mediated by the extracellular domain) while the small membrane-spanning fragment of JAM-A which remains after cleavage might allow intracellular signaling that promotes tumorigenic processes, including proliferation [17], migration [14, 15], invasion [14], and apoptosis inhibition [16]. Furthermore, it is possible that JAM-A cleavage generates a constitutively activated truncated form of the protein, analogous to other transmembrane proteins, including HER2 [37, 50, 51]. This could account for observed associations between JAM-A cleavage and resistance to anti-HER2 therapy. However, although our data suggest that cleaved JAM-A may play more of a role in regulating cellular invasion while the small membrane-spanning fragment left after cleavage is involved in cell viability, it is not known at this point whether cJAM-A release is directly responsible for observed events such as the upregulation of EGFR in treatment-resistant cells. It may be the case that residual membranous JAM-A stabilizes or upregulates EGFR expression and that JAM-A cleavage merely accompanies this event. Regardless, this will be an interesting future avenue to pursue, particularly since signaling from JAM-A and other tight junction proteins is emerging as a key player in many processes even outside the realm of cell–cell adhesion [20, 52].

Another contribution to tumorigenicity potentially associated with reduced adhesive function (secondary to JAM-A cleavage) reflects the integral role of tight junctions in maintaining normal breast epithelial architectural integrity. Architectural disruption itself could promote local invasion/migration, facilitating downstream metastasis. Several studies have reported a role for JAM-A signaling in breast cancer cell migration [14, 15, 44]. Our study has provided the first evidence that a mimetic of cleaved JAM-A promotes breast cancer cell invasion *in vitro* and in a semi-*in vivo* model in addition to proliferation in the latter*.* It is intriguing to speculate as to why rcJAM-A did not stimulate proliferation *in vitro* as it did in the semi-*in vivo* model. Although it is possible that cJAM-A acts differently from membranous JAM-A in altering functional cellular behaviors, an alternative explanation is that perhaps the positive effects of cJAM-A on invasion are exerted directly on migrating epithelial cells but that its pro-proliferative effect requires other auxiliary cell types which would have been present in the semi-*in vivo* model but not in isolated cell cultures. As to how cJAM-A might mechanistically drive biological signaling mechanisms, some preliminary evidence suggests that up- or downregulated phosphorylation of different members related to the MAPK family of kinases might play a role (Additional file 5). This suggests a mechanism whereby JAM-A cleavage promotes breast cancer cell migration/invasion through (1) preservation of JAM-A–dependent intracellular signaling, (2) loss of intercellular adhesion, and (3) extracellular release of invasion-promoting cJAM-A. This is supported by the association between JAM-A overexpression and metastatic disease at diagnosis in breast tumors of patients who developed disease recurrence following anti-HER2 therapy. It is intriguing to speculate that inhibiting JAM-A limits the invasive/migratory potential of HER2-positive breast cancer cells with innate or acquired resistance to anti-HER2 therapy.

In addition, the association between JAM-A cleavage and resistance to trastuzumab and lapatinib may facilitate monitoring of breast tumor sensitivity to anti-HER2 treatment. Our results have provided the first evidence of cJAM-A detection in the serum of patients with HER2-positive breast cancer, and excitingly our work shows for the first time that levels of cJAM-A are significantly higher in patients who developed a recurrence while on HER2-targeted therapy. Although a larger cohort of patient samples (preferably at multiple sites) will be important for verification, it suggests that cJAM-A may represent a novel biomarker enabling prospective identification of HER2-positive patients at greatest risk of developing therapeutic resistance.

Given the observed associations between JAM-A cleavage and drug resistance, it might be the case that overexpression of both JAM-A and particularly ADAM10 is required for the acquisition of resistance to anti-HER2 therapies. This hypothesis is supported by results revealing that silencing ADAM10 or ADAM17 (or both) only partially re-sensitized trastuzumab- and lapatinib-resistant cells to anti-HER2 therapy. It is further supported by IHC from a small patient cohort of 34 patients in which 100% (7 out of 7) of the patients who developed disease recurrence following anti-HER2 treatment expressed high levels of JAM-A. Admittedly, 81.5% of the patients whose tumors had not recurred up to the time of analysis also had high levels of JAM-A but it cannot be excluded that some of those patients may have gone on to experience recurrence soon after our analysis. The more important point is that nobody who developed a recurrence in our small pilot study had low levels of JAM-A. Returning to the role of ADAM10, previous studies have shown that higher pre-treatment expression of ADAM10 in breast tumors correlates with decreased clinical response of patients to trastuzumab and poorer relapse-free survival [47]. In our hands, ADAM10 had a greater relationship with JAM-A cleavage than ADAM17. Thus, IHC staining of JAM-A and ADAM10 may help identify patients at high risk of developing resistance. However, upregulation of ADAM10 has also been shown to occur in response to anti-HER2 treatment throughout development of resistance to therapy [47]. Therefore, resistance-associated overexpression of ADAM10 may not be detectable at diagnosis. Nonetheless, overexpression of JAM-A alone may be indicative of potential to acquire resistance to anti-HER2 therapy. Even more valuable may be combinatorial measurements of cJAM-A levels (by ELISA) in serum correlated with ADAM10 expression levels (by IHC) on patient tissue sections. Furthermore, it is noteworthy that silencing JAM-A reduced the viability of drug-sensitive cell lines as well as those resistant to anti-HER2 therapy (data not shown). Thus, dual JAM-A/HER2 targeting may be of benefit as a neo-adjuvant treatment of HER2-positive patients with as well as those who acquire resistance to anti-HER2 therapy. This is supported by studies demonstrating pro-tumorigenic roles for JAM-A [17–19]. Although our study is the first to demonstrate a potential link between JAM-A expression/cleavage and resistance to the HER2-targeted therapies trastuzumab and lapatinib, it will be interesting in future studies to explore whether the same mechanism plays any role in the development of resistance to newer targeted therapies such as the dual HER2/HER3 inhibitory monoclonal antibody pertuzumab. This is particularly relevant to emerging cancer combination therapies since pertuzumab has been shown to have a significant benefit when combined with trastuzumab and chemotherapy [12].

## Conclusions

We have shown that, in breast cancer cellular models, JAM-A levels correlate directly with HER2 and EGFR expression, phosphorylation of Akt, and viability of cells with resistance to anti-HER2 therapy. It is not currently known whether overexpression of JAM-A by itself would be sufficient to induce resistance to HER2-targeted drugs. In the setting of short-term drug treatments *in vitro*, JAM-A overexpression alone was unable to enhance cell viability (Additional file 6). However, this is entirely consistent with the development of drug resistance in cell lines being a complex and multi-factorial process which occurs over months rather than days [54, 55]. Our focus in this article was to try to understand what the consequences of such overexpression might be and whether it could be linked to the detection or pathophysiology of recurrent disease. In this respect, it was a crucial finding that cleavage of the JAM-A extracellular domain (which would be increasingly likely in JAM-overexpressing settings) is associated with resistance to HER2-targeted therapies and that cleaved JAM-A promotes the invasive potential of breast cancer cells *in vitro* and in a semi-*in vivo* model. Importantly, we used two separate HER2-positive cell lines (one luminal B subtype and one HER2-positive subtype) and thus are confident that our findings in relation to JAM/HER2 crosstalk and the relevance of JAM-A cleavage to drug resistance were not an artefact of one cell line or breast cancer molecular subtype. Even more importantly, our *in vitro* work was followed up by pilot translational studies in patient samples. Specifically, analysis of JAM-A tumor expression levels in a small cohort of patients with HER2-positive breast cancer has suggested links between JAM-A expression and the development of resistance to anti-HER2 therapies. Additionally, JAM-A overexpression significantly correlated with metastatic disease at diagnosis in a cohort of 70 patients with aggressive trastuzumab-resistant breast cancer. Lastly, a pilot study revealed that cleaved JAM-A is detectable in serum of patients with HER2-positive breast cancer and shows a correlation with resistance to anti-HER2 therapies. Our results thus far strongly support the viability of future investigations into the role of JAM-A in resistance to anti-HER2 therapies and may reveal mechanistic insights into the development of resistance itself in addition to providing a novel pharmacological target or biomarker of drug resistance in patients with HER2-positive breast cancer.

## Additional files

Additional file 1: Lapatinib treatment significantly reduces viability of SK-BR-3–sensitive but not SK-BR-3 lapatinib-resistant cells. SK-BR3–sensitive and lapatinib-resistant cells (1500) were plated in triplicate wells of 96-well plates and treated the following day with the highest concentration of vehicle control (VC) (dimethyl sulfoxide, 0.002% vol/vol) or the stated concentration of lapatinib; 72 h later, cellular viability was measured via MTT (3-(4,5-dimethylthiazol-2-yl)-2,5-diphenyltetrazolium bromide) assay. (A) Cell viability response of SKBR3-sensitive cells to a range of lapatinib treatments. (B) Cell viability response of SKBR3 lapatinib-resistant cells to a range of lapatinib treatments. Lapatinib treatment significantly reduced SKBR3-sensitive cell viability but not that of SKBR3 lapatinib-resistant cells. *P <0.05, **P <0.01, ***P <0.001 by one-way analysis of variance with Tukey’s multiple comparison test, *n* = 3 independent experiments. (TIF 42 kb)

Additional file 2: The functional effects of JAM-A silencing are reproduced using an alternative small interfering RNA (siRNA). (A) MCF7-HER2 cells were plated at 150,000 cells per well in six-well plates and transfected the following day with 25 nM of control siRNA (siNEG; D-001810-01-05, Dharmacon) or JAM-A siRNA (siJAM-A2;CGGGGGUCGCAGGAAUCUGUU, Dharmacon); 72 h later, protein was extracted for Western blot analysis. JAM-A knockdown using an alternative siRNA significantly reduced JAM-A protein levels. In addition, HER2 protein levels were concurrently reduced in these conditions. Densitometric analysis shows HER2 expression normalized to actin as a loading control. **P <0.01 by equal variance unpaired *t* test, *n* = 3 independent experiments. (B) 1500 cells per well of trastuzumab-resistant BT-474 and SK-BR-3 cells were plated in triplicate on 96-well plates and transfected the following day with 25 nM of control or JAM-A siRNA (as above); 24 h later, cells were treated with vehicle control (VC; sterile nuclease-free water, 0.5% vol/vol) or trastuzumab (100 μg/mL or 10 μg/mL for BT474 trastuzumab-resistant and SKBR3 trastuzumab-resistant cells, respectively); 72 h later, cell viability was measured via MTT (3-(4,5-dimethylthiazol-2-yl)-2,5-diphenyltetrazolium bromide) assay. Silencing JAM-A expression in addition to anti-HER2 treatment was more effective than anti-HER2 treatment alone at reducing cell viability. *P <0.05, **P <0.01, ***P <0.001 by one-way analysis of variance with Tukey’s multiple comparison test, n = 3 independent experiments. (TIF 111 kb)

Additional file 3: A disintegrin and metalloproteinase (ADAM) inhibition does not have an additive effect with anti-HER2 treatment in drug-resistant cell lines. Trastuzumab-resistant BT-474 cells and lapatinib-resistant SK-BR-3 cells were plated at 1500 cells per well in 96-well plates; 24 h later, cells were treated with either vehicle control (VC) (dimethyl sulfoxide (DMSO), 0.3% vol/vol) or the ADAM inhibitor GI254023X (GI25; 12 μg/mL; SML0789, Sigma-Aldrich). The following day, trastuzumab-resistant BT474 cells were treated with VC (sterile nuclease-free water, 0.5% vol/vol) or 100 μg/mL trastuzumab- and lapatinib-resistant SKBR3 cells were treated with VC (DMSO, 0.002% vol/vol) or 250 nM lapatinib; 72 h later, cell viability was measured via MTT (3-(4,5-dimethylthiazol-2-yl)-2,5-diphenyltetrazolium bromide) assay. (A) Cell viability response of BT-474 trastuzumab-resistant cells to trastuzumab treatment alone and combined with GI25 treatment. (B) Cell viability response of SK-BR-3 lapatinib-resistant cells to lapatinib treatment alone and combined with GI25 treatment. ADAM inhibition alone significantly reduced cell viability of BT-474 trastuzumab-resistant cells and SK-BR-3 lapatinib-resistant cells but did not have an additive effect with anti-HER2 treatment. *P <0.05, **P <0.01, ***P <0.001 by one-way analysis of variance with Tukey’s multiple comparison test, *n* = 3 independent experiments. (TIF 66 kb)

Additional file 4: Recombinant soluble JAM-A treatment does not affect the viability or colony-forming ability of drug-sensitive breast cancer cells. (A, B) Trastuzumab-sensitive BT474 and lapatinib-sensitive SKBR3 cells were plated at 1500 cells per well in 96-well plates. The following day, cells were treated in serum-free media with vehicle control (phosphate-buffered saline (PBS), 0.0004% vol/vol for BT-474–sensitive and 0.0001% vol/vol for SK-BR-3–sensitive) or specified concentrations of recombinant cleaved (soluble) JAM-A (rcJAM-A; Recombinant Human Junctional Adhesion Molecule 1 protein, ab151859, Abcam). Specified concentrations of rcJAM-A were selected on the basis of previously described approximation of cJAM-A levels naturally released by corresponding drug-resistant cells; 72 h later, cell viability was measured via MTT (3-(4,5-dimethylthiazol-2-yl)-2,5-diphenyltetrazolium bromide) assay. Cell viability response of trastuzumab-sensitive BT-474 (A) and lapatinib-sensitive SK-BR-3 cells (B) to recombinant soluble JAM-A treatment. Recombinant soluble JAM-A treatment did not affect the viability of either cell line. Quantitative analysis is based on n = 3 independent experiments. (C, D) Lapatinib-sensitive SKBR3 cells were plated at 15,000 cells per well in six-well plates. The following day, cells were treated with 0.5 ng/mL rcJAM-A, 1 ng/mL rcJAM-A, or PBS as vehicle control. Cells were retreated twice at 72 h intervals. After 9 days of treatment, colonies were fixed and stained with crystal violet. Recombinant soluble JAM-A treatment had no effect on colony-forming potential of SKBR3-sensitive cells. Quantitative analysis of colony number is based on n = 3 independent experiments. (TIF 178 kb)

Additional file 5: Alterations in mitogen-activated protein kinase (MAPK) signaling following treatment of breast cancer cells with cJAM-A. Lapatinib-sensitive SK-BR-3 cells on six-well plates were treated for 72 h with 1 ng/mL recombinant cleaved JAM-A (rcJAM-A) or left untreated for the same period. Lysates were then exposed to a phospho-MAPK array in accordance with the instructions of the manufacturer (R&D Systems, ARY002B). Spots were developed by enhanced chemiluminescence and captured on a ChemiDoc analyzer (Bio-Rad). Differences in spot intensity between experimental conditions were quantitatively compared using ImageJ software. Relative to untreated controls, phosphorylation of three targets each significantly increased or decreased. (*P <0.05, **P <0.01 by two-tailed unpaired Student’s *t* test.) (TIF 105 kb)

Additional file 6: JAM-A overexpression is insufficient to improve cell viability following short-term treatment with HER2-targeted drugs. MDA-MB-231 cells overexpressing full-length human JAM-A (JAM+) or a pcDNA3 empty vector control (EV) were treated for 3 days in serum-free medium with the indicated concentrations of trastuzumab (A) or lapatinib (B) and subjected to MTT (3-(4,5-dimethylthiazol-2-yl)-2,5-diphenyltetrazolium bromide) viability assays. In parallel, the same cell lines were treated for 6 days in complete medium with the indicated concentrations of trastuzumab (C) or lapatinib (D) and subjected to MTT viability assays. JAM-A overexpression did not alter the drug sensitivity of cells under any of the short-term conditions tested. (E) Representative protein overexpression of JAM-A in transfected MDA-MB-231 cells. (TIF 145 kb)

## Abbreviations

ADAM: A disintegrin and metalloproteinase
BCA: Bicinchoninic acid assay
CAM: Chorioallantoic membrane
cJAM-A: Cleaved JAM-A
DMSO: Dimethyl sulfoxide
EGFR: Epidermal growth factor receptor
ELISA: Enzyme-linked immunosorbent assay
FBS: Fetal bovine serum
HER2: Human epidermal growth factor receptor-2
IHC: Immunohistochemistry
JAM-A: Junctional adhesion molecule-A
MAPK: Mitogen-activated protein kinase
MTT: 3-(4,5-dimethylthiazol-2-yl)-2,5-diphenyltetrazolium bromide
PBS: Phosphate-buffered saline
PI3K: Phosphoinositide-3-kinase
qPCR: Quantitative polymerase chain reaction
rcJAM-A: Recombinant cleaved JAM-A
RT-qPCR: Reverse transcription–quantitative polymerase chain reaction
siRNA: Small interfering RNA
