# Supplementary figures and images for: Cleavage of the extracellular domain of junctional adhesion molecule-A is associated with resistance to anti-HER2 therapies in breast cancer settings
Source: Breast Cancer Res. 2018 Nov 20;20:140. doi: 10.1186/s13058-018-1064-1 (PMC6247757; doi:10.1186/s13058-018-1064-1)

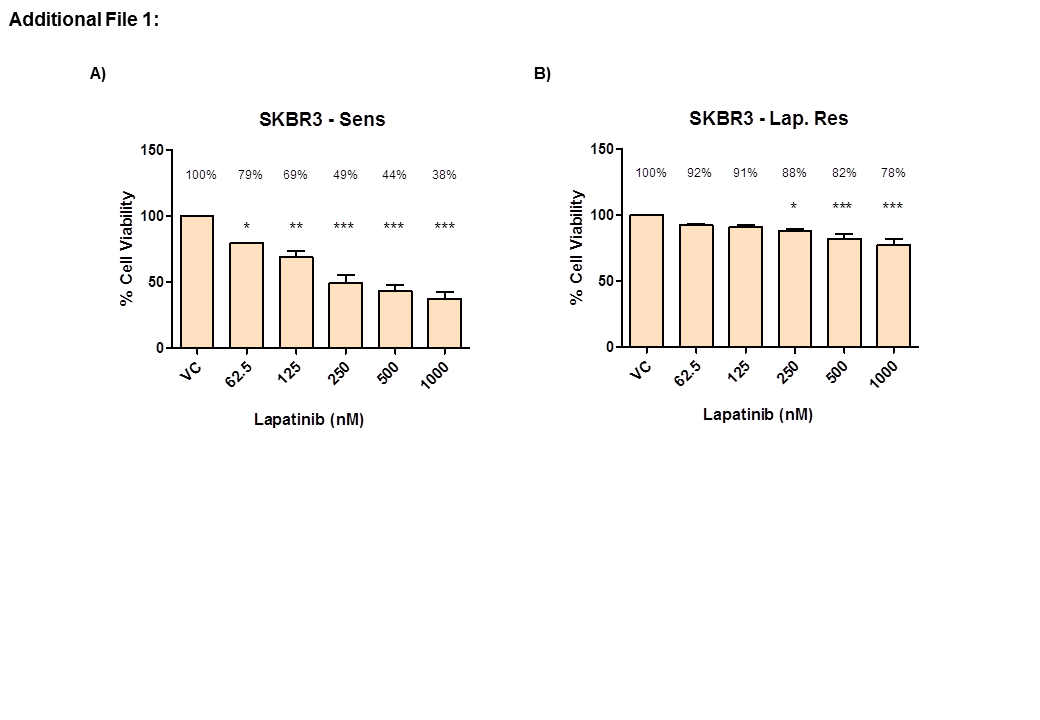

Supplement: Supplementary file 1 — Lapatinib treatment significantly reduces viability of SK-BR-3–sensitive but not SK-BR-3 lapatinib-resistant cells. SK-BR3–sensitive and lapatinib-resistant cells (1500) were plated in triplicate wells of 96-well plates and treated the following day with the highest concentration of vehicle control (VC) (dimethyl sulfoxide, 0.002% vol/vol) or the stated concentration of lapatinib; 72 h later, cellular viability was measured via MTT (3-(4,5-dimethylthiazol-2-yl)-2,5-diphenyltetrazolium bromide) assay. (A) Cell viability response of SKBR3-sensitive cells to a range of lapatinib treatments. (B) Cell viability response of SKBR3 lapatinib-resistant cells to a range of lapatinib treatments. Lapatinib treatment significantly reduced SKBR3-sensitive cell viability but not that of SKBR3 lapatinib-resistant cells. *P <0.05, **P <0.01, ***P <0.001 by one-way analysis of variance with Tukey’s multiple comparison test, n = 3 independent experiments. (TIF 42 kb) [file 13058_2018_1064_MOESM1_ESM.tif]

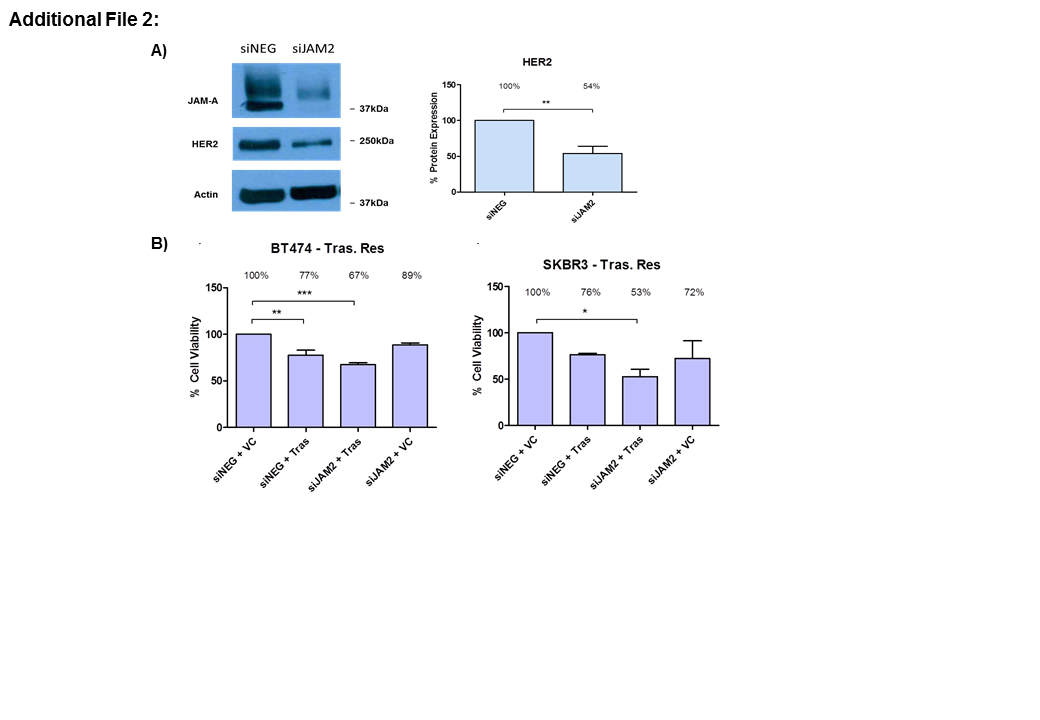

Supplement: Supplementary file 2 — The functional effects of JAM-A silencing are reproduced using an alternative small interfering RNA (siRNA). (A) MCF7-HER2 cells were plated at 150,000 cells per well in six-well plates and transfected the following day with 25 nM of control siRNA (siNEG; D-001810-01-05, Dharmacon) or JAM-A siRNA (siJAM-A2;CGGGGGUCGCAGGAAUCUGUU, Dharmacon); 72 h later, protein was extracted for Western blot analysis. JAM-A knockdown using an alternative siRNA significantly reduced JAM-A protein levels. In addition, HER2 protein levels were concurrently reduced in these conditions. Densitometric analysis shows HER2 expression normalized to actin as a loading control. **P <0.01 by equal variance unpaired t test, n = 3 independent experiments. (B) 1500 cells per well of trastuzumab-resistant BT-474 and SK-BR-3 cells were plated in triplicate on 96-well plates and transfected the following day with 25 nM of control or JAM-A siRNA (as above); 24 h later, cells were treated with vehicle control (VC; sterile nuclease-free water, 0.5% vol/vol) or trastuzumab (100 μg/mL or 10 μg/mL for BT474 trastuzumab-resistant and SKBR3 trastuzumab-resistant cells, respectively); 72 h later, cell viability was measured via MTT (3-(4,5-dimethylthiazol-2-yl)-2,5-diphenyltetrazolium bromide) assay. Silencing JAM-A expression in addition to anti-HER2 treatment was more effective than anti-HER2 treatment alone at reducing cell viability. *P <0.05, **P <0.01, ***P <0.001 by one-way analysis of variance with Tukey’s multiple comparison test, n = 3 independent experiments. (TIF 111 kb) [file 13058_2018_1064_MOESM2_ESM.tif]

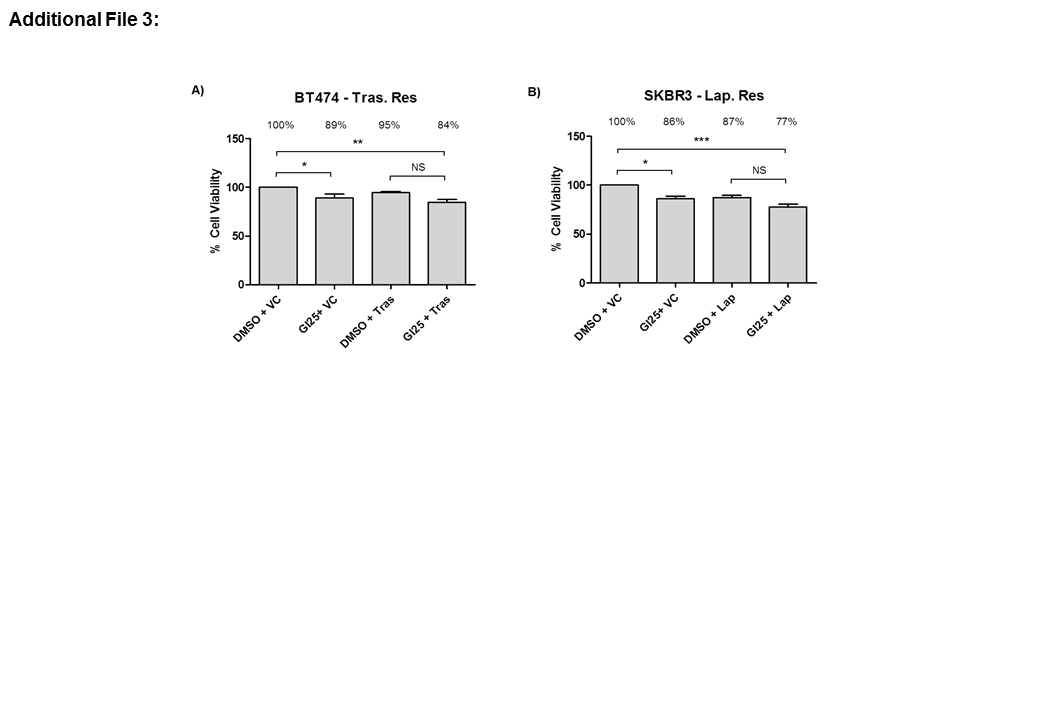

Supplement: Supplementary file 3 — A disintegrin and metalloproteinase (ADAM) inhibition does not have an additive effect with anti-HER2 treatment in drug-resistant cell lines. Trastuzumab-resistant BT-474 cells and lapatinib-resistant SK-BR-3 cells were plated at 1500 cells per well in 96-well plates; 24 h later, cells were treated with either vehicle control (VC) (dimethyl sulfoxide (DMSO), 0.3% vol/vol) or the ADAM inhibitor GI254023X (GI25; 12 μg/mL; SML0789, Sigma-Aldrich). The following day, trastuzumab-resistant BT474 cells were treated with VC (sterile nuclease-free water, 0.5% vol/vol) or 100 μg/mL trastuzumab- and lapatinib-resistant SKBR3 cells were treated with VC (DMSO, 0.002% vol/vol) or 250 nM lapatinib; 72 h later, cell viability was measured via MTT (3-(4,5-dimethylthiazol-2-yl)-2,5-diphenyltetrazolium bromide) assay. (A) Cell viability response of BT-474 trastuzumab-resistant cells to trastuzumab treatment alone and combined with GI25 treatment. (B) Cell viability response of SK-BR-3 lapatinib-resistant cells to lapatinib treatment alone and combined with GI25 treatment. ADAM inhibition alone significantly reduced cell viability of BT-474 trastuzumab-resistant cells and SK-BR-3 lapatinib-resistant cells but did not have an additive effect with anti-HER2 treatment. *P <0.05, **P <0.01, ***P <0.001 by one-way analysis of variance with Tukey’s multiple comparison test, n = 3 independent experiments. (TIF 66 kb) [file 13058_2018_1064_MOESM3_ESM.tif]

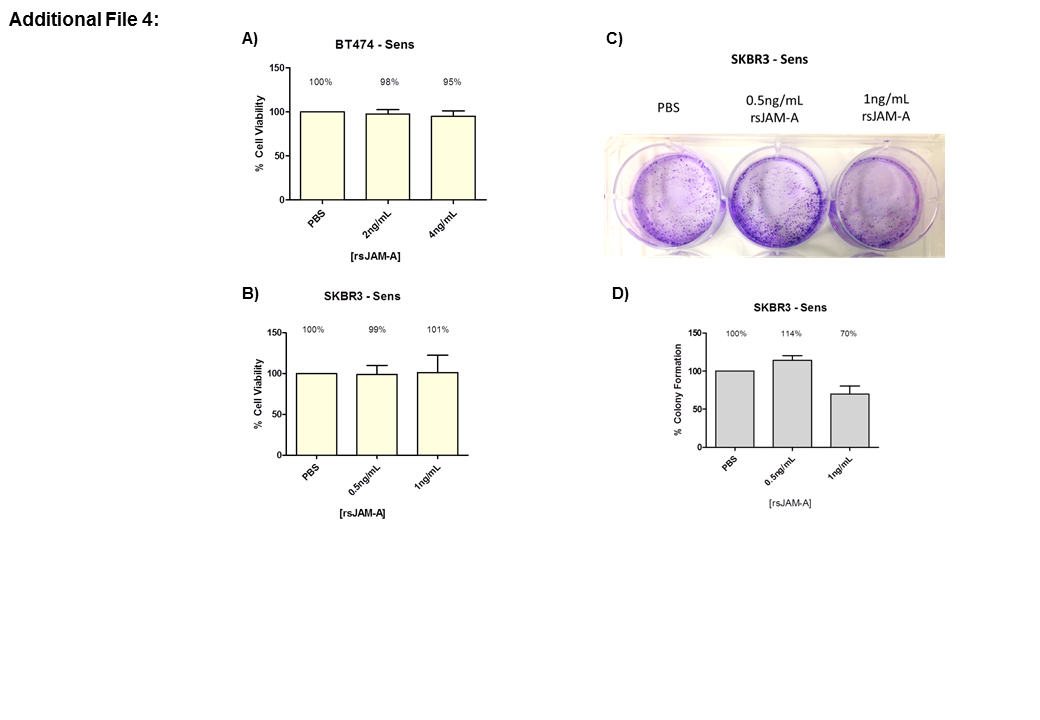

Supplement: Supplementary file 4 — Recombinant soluble JAM-A treatment does not affect the viability or colony-forming ability of drug-sensitive breast cancer cells. (A, B) Trastuzumab-sensitive BT474 and lapatinib-sensitive SKBR3 cells were plated at 1500 cells per well in 96-well plates. The following day, cells were treated in serum-free media with vehicle control (phosphate-buffered saline (PBS), 0.0004% vol/vol for BT-474–sensitive and 0.0001% vol/vol for SK-BR-3–sensitive) or specified concentrations of recombinant cleaved (soluble) JAM-A (rcJAM-A; Recombinant Human Junctional Adhesion Molecule 1 protein, ab151859, Abcam). Specified concentrations of rcJAM-A were selected on the basis of previously described approximation of cJAM-A levels naturally released by corresponding drug-resistant cells; 72 h later, cell viability was measured via MTT (3-(4,5-dimethylthiazol-2-yl)-2,5-diphenyltetrazolium bromide) assay. Cell viability response of trastuzumab-sensitive BT-474 (A) and lapatinib-sensitive SK-BR-3 cells (B) to recombinant soluble JAM-A treatment. Recombinant soluble JAM-A treatment did not affect the viability of either cell line. Quantitative analysis is based on n = 3 independent experiments. (C, D) Lapatinib-sensitive SKBR3 cells were plated at 15,000 cells per well in six-well plates. The following day, cells were treated with 0.5 ng/mL rcJAM-A, 1 ng/mL rcJAM-A, or PBS as vehicle control. Cells were retreated twice at 72 h intervals. After 9 days of treatment, colonies were fixed and stained with crystal violet. Recombinant soluble JAM-A treatment had no effect on colony-forming potential of SKBR3-sensitive cells. Quantitative analysis of colony number is based on n = 3 independent experiments. (TIF 178 kb) [file 13058_2018_1064_MOESM4_ESM.tif]

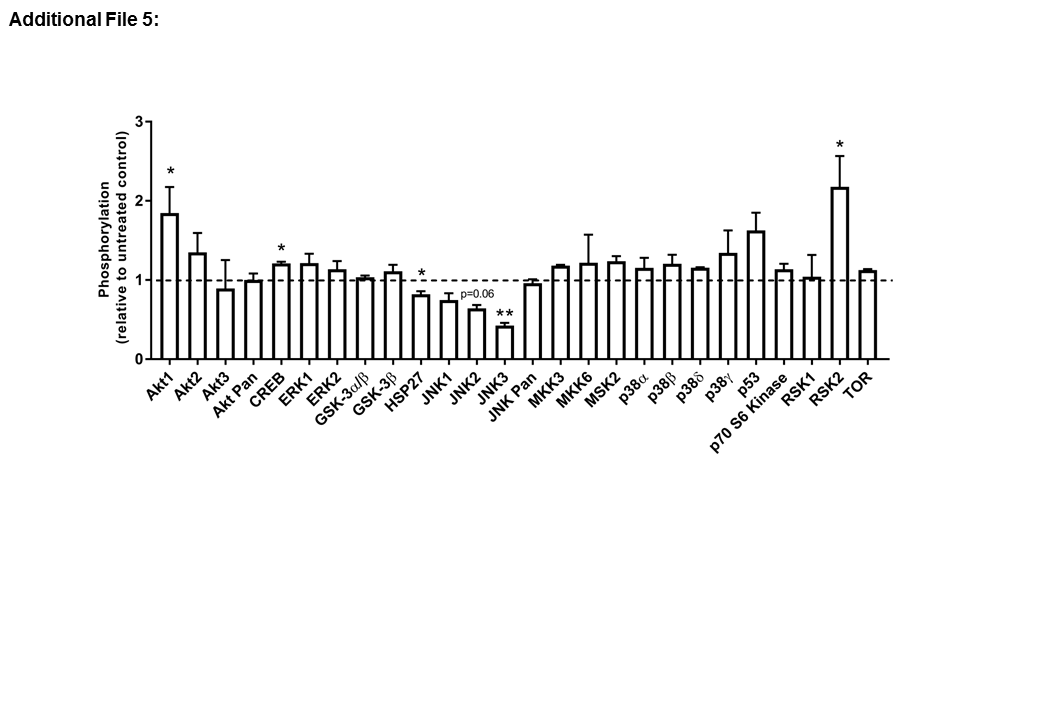

Supplement: Supplementary file 5 — Alterations in mitogen-activated protein kinase (MAPK) signaling following treatment of breast cancer cells with cJAM-A. Lapatinib-sensitive SK-BR-3 cells on six-well plates were treated for 72 h with 1 ng/mL recombinant cleaved JAM-A (rcJAM-A) or left untreated for the same period. Lysates were then exposed to a phospho-MAPK array in accordance with the instructions of the manufacturer (R&D Systems, ARY002B). Spots were developed by enhanced chemiluminescence and captured on a ChemiDoc analyzer (Bio-Rad). Differences in spot intensity between experimental conditions were quantitatively compared using ImageJ software. Relative to untreated controls, phosphorylation of three targets each significantly increased or decreased. (*P <0.05, **P <0.01 by two-tailed unpaired Student’s t test.) (TIF 105 kb) [file 13058_2018_1064_MOESM5_ESM.tif]

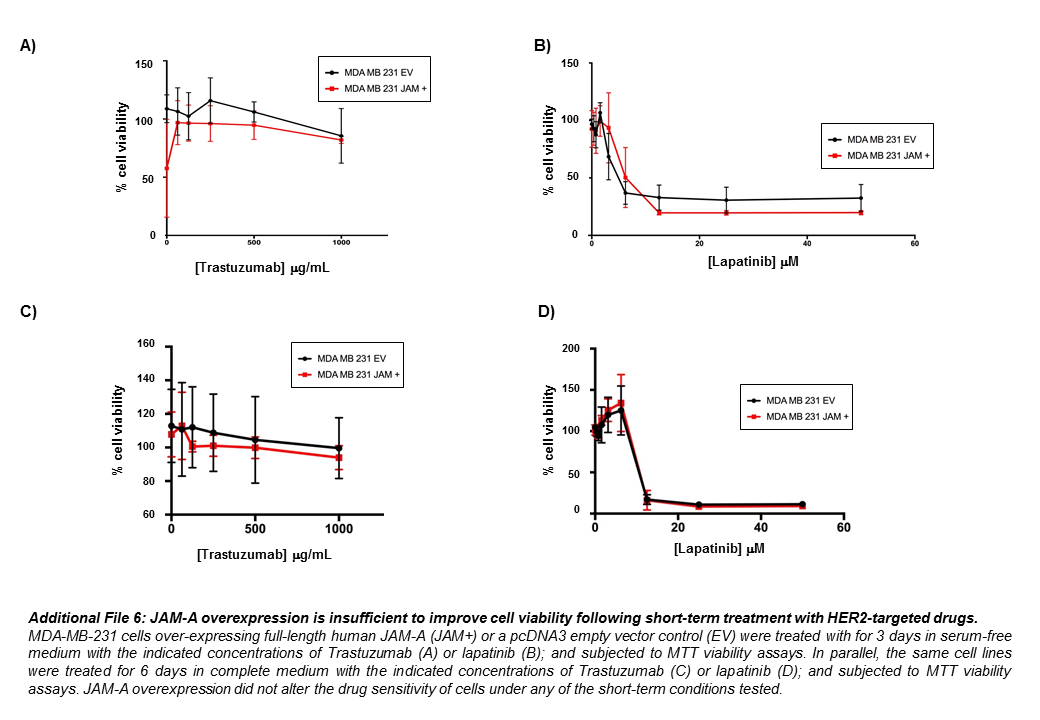

Supplement: Supplementary file 6 — JAM-A overexpression is insufficient to improve cell viability following short-term treatment with HER2-targeted drugs. MDA-MB-231 cells overexpressing full-length human JAM-A (JAM+) or a pcDNA3 empty vector control (EV) were treated for 3 days in serum-free medium with the indicated concentrations of trastuzumab (A) or lapatinib (B) and subjected to MTT (3-(4,5-dimethylthiazol-2-yl)-2,5-diphenyltetrazolium bromide) viability assays. In parallel, the same cell lines were treated for 6 days in complete medium with the indicated concentrations of trastuzumab (C) or lapatinib (D) and subjected to MTT viability assays. JAM-A overexpression did not alter the drug sensitivity of cells under any of the short-term conditions tested. (E) Representative protein overexpression of JAM-A in transfected MDA-MB-231 cells. (TIF 145 kb) [file 13058_2018_1064_MOESM6_ESM.tif]
